# Book Reviews

**Published:** 1872-05-01

**Authors:** 


### Book Reviews.

Venesection, its Abuse Formerly—its Neglect at the Present Day. A paper read to the Massachusetts Medical Society, June 6th, 1871. By Henry I. Bowditch, M. D., of Boston, Mass.

This is a pamphlet of 33 pages, in which the author presents briefly the true, or rather correct, view in regard to the value of Venesection as a therapeutic agent in the treatment of disease, and illustrates the subject by the detail of four interesting cases. The following are the conclusions of the writer in regard to the principal classes of cases in which bleeding is likely to prove beneficial.

*First*—In all acute or chronic cases where from any cause the heart apparently becomes distended with blood and consequently the circulation is greatly impeded, whereby orthopnœa, lividity of the skin, with a very feeble, small, and generally rapid pulse, mixed perhaps with other serious and distressing symptoms, are produced.

*Second*—It seems not uncalled for when there is a very acute pain in any part of the thorax, for example, from inflammation of the pleura, causing orthopnœa and distress, even if there be no obstruction of the heart's action. The question has often arisen in my mind whether the many cases of large pleuritic effusions which we see now-a-days, may not be due, at least in part, to our neglect of venesection during the earlier and more acute period of the disease—when pain in the side is quite severe, and all the symptoms are very marked and the pulse and temperature are increased.

*Third*—In violent acute cephalic symptoms, threatening serious results, when the head is hot, the face flushed, and the pulse is full and hard.

*Fourth*—In certain cases of threatened miscarriage, occurring at certain times during pregnancy, I know of nothing better than venesection to check the tendency. Even after hemorrhage from the womb had actually commenced, and even when it was attended with expulsive action of the uterus, I had, many years ago, a case wholly relieved by venesection to the amount of a few ounces only.

Lectures on Aural Catarrh; or the Commonest forms of Deafness, and their Cure. Mostly delivered at St. Mary's Hospital, Edinburgh. By Peter Allen, M.D., Fellow of Royal College of Surgeons, Ed.; Aural Surgeon to, and Lecturer at St. Mary's Hospital, etc., etc. New York; William Wood & Co., 27 Great Jones street. 1872.

This is a small sized octavo volume of 228 pages, published in excellent style, including paper, type, and illustrations. The diseases treated of are of very frequent occurrence, highly important so far as regards the comfort of the patients, and yet as little understood by the general practitioner as any class of ailments that could be named. We think this work will be found convenient and valuable by all classes of practitioners. Received from the publishers through the bookstore of Wm. B. Keen, Cooke & Co., Chicago.

The Treatment of Venereal Diseases: A Monograph on the Method pursued in the Vienna Hospital, under the direction of Prof. Von Sigmund, including all the formulæ. By M. H. Henry, M.D., Surgeon to the New York Dispensatory, Department of Venereal and Skin Diseases; Fellow of the New York Academy of Medicine, etc., etc., etc. Adapted and arranged from the German. New York: William Wood & Co., 27 Great Jones street. 1872. Received through the bookstore of Wm. B. Keen, Cooke & Co., Chicago.

This is a monograph, published in good style, and well bound, containing 49 pages; the first fifteen of which are occupied with general directions and explanations, and the remaining part of the work with particular formulas or prescriptions, of which there are a great variety, intended to meet almost every form and variety of syphilitic disease.

---

Spasmodic Muscular Contraction—Arterial Compression.—M. Broca had under his care, a few months ago, in the Hospital de la Petie, a man who had broken both bones of his legs an hour before his admission to the hospital. The muscular contraction was so violent that it was impossible to reduce the fracture. M. Broca thereon employed a method which he had found successful in cases of painful cramp of the lower limbs, viz., compression of the femoral artery. Almost immediately the muscles became relaxed, and reduction was effected with ease. Subsequently, in re-applying the splints, the contraction returned, and was overcome by the same means.—*Medical Record.*

